# A Retrospective Comparative Evaluation of Selected Blood Cell Ratios, Acute Phase Proteins, and Leukocyte Changes Suggestive of Inflammation in Cats

**DOI:** 10.3390/ani13162579

**Published:** 2023-08-10

**Authors:** Giulia Donato, Maria Grazia Pennisi, Maria Flaminia Persichetti, Joy Archer, Marisa Masucci

**Affiliations:** 1 Department of Veterinary Sciences, University of Messina, 98168 Messina, Italy; mariagrazia.pennisi@unime.it (M.G.P.); mfpersichetti@gmail.com (M.F.P.); 2 Department of Veterinary Medicine, University of Cambridge, Madingley Road, Cambridge CB3 0ES, UK; ja331@cam.ac.uk

**Keywords:** neutrophil-to-lymphocyte ratio, monocyte-to-lymphocyte ratio, platelet-to-lymphocyte ratio, leukocyte toxic changes, serum amyloid A, albumin, globulins, albumin-to-globulin ratio, inflammation, cat

## Abstract

**Simple Summary:**

During inflammation, acute-phase proteins or leukocyte toxic changes may be observed in dogs and cats, aiding in the diagnosis and prognosis of inflammatory diseases. Leukocyte ratios are generally evaluated in human medicine for the diagnosis and prognosis of inflammatory and neoplastic conditions. Recently, some of these ratios have been evaluated in dogs and cats, showing useful results; however, their relationship to routinely measured markers of inflammation has never been investigated in cats. The present study showed changes in neutrophil-to-lymphocyte (NLR), monocyte-to-lymphocyte (MLR), and platelet-to-lymphocyte (PLR) ratios correlated with those of other markers of inflammation in cats, such as serum amyloid A, albumin, globulins, and albumin-to-globulin ratio. The values of NLR and MLR in cats with no changes in parameters indicative of inflammation were significantly lower when compared with those of cats with increased SAA or hypoalbuminemia. These results demonstrate that leukocyte ratios can be valuable markers of inflammation in cats.

**Abstract:**

Neutrophil-to-lymphocyte (NLR), monocyte-to-lymphocyte (MLR), and platelet-to-lymphocyte (PLR) ratios have been proposed as diagnostic and prognostic markers for neoplastic and inflammatory diseases in dogs and cats. The aim of this retrospective preliminary study was to evaluate the relationship between these ratios and markers of inflammation routinely measured in cats. A total of 275 cats were enrolled. Complete blood count, serum amyloid A (SAA), albumin, globulin, and albumin-to-globulin ratio (AGR) data were analyzed, as well as the presence of leukocyte alterations considered suggestive of inflammation (LAI: neutrophils left shift, toxic neutrophils, and reactive lymphocytes) evaluated in blood smears. The NLR and MLR correlated positively with SAA and globulins and negatively with albumin and AGR. Higher NLR and MLR were found in cats with increased SAA and globulins and decreased albumin and AGR. The PLR correlated negatively with albumin and AGR. A higher PLR was found in cats with hypoalbuminemia. Cats with LAI had higher NLR, MLR, and PLR. In cats with no changes in parameters indicative of inflammation, 11.25, 0.42, and 528.3 were identified as upper limits for NLR, MLR, and PLR, respectively. In conclusion, the NLR, MLR, and PLR act as good inflammatory markers easily evaluated by routine hematology.

## 1. Introduction

White blood cells play a primary and complex role in the systemic inflammatory response associated with both infectious and non-infectious causative agents, with changes in morphology and count of circulating leukocyte populations [1,2,3,4,5]. In fact, cytokines released during inflammation stimulate the hypothalamic-pituitary-adrenal axis with the rapid release of mature neutrophils from the marginal pool, resulting in neutrophilia [6]. At the same time, bone marrow production and release of neutrophils are stimulated, and, in cases of severe inflammation, neutrophil precursors (mostly band neutrophils) are also released from the bone marrow [6]. Moreover, monocyte counts can increase in cases of inflammation associated with infection or necrosis [4,5]. Lymphopenia is a hallmark of stress and may also be observed during inflammation as a result of margination and redistribution of lymphocytes within lymphoid organs and excessive apoptosis [2]. In addition to changes in the leukocyte population, cytokines released by inflammatory cells stimulate thrombopoiesis, and increased platelet counts can be observed [7].

Prognostic markers are of great clinical interest in both human and veterinary medicine, and in recent years, the accuracy of blood cell ratios as cost-effective and easily accessible diagnostic and prognostic markers of various inflammatory conditions has been investigated. Both the neutrophil-to-lymphocyte ratio (NLR) and monocyte-to-lymphocyte ratio (MLR) represent the balance between innate (neutrophils and monocytes) and adaptive (lymphocytes) immune responses. These white blood cell populations are involved in the patho-mechanisms of inflammation and stress disorders at the same time [8,9]. In humans, NLR, MLR, and platelet-to-lymphocyte ratio (PLR) values were studied as inflammatory markers in various conditions for diagnostic and prognostic purposes [2,8,10,11,12,13,14]. High values of NLR and MLR were considered accurate additional markers in the diagnosis of acute coronary syndrome [8]. The NLR value was found to be positively correlated with the severity of the clinical course in critically ill patients [2], and increased values were associated with a higher risk of recurrence of aneurysmal bone cysts [12]. In patients with lymphadenopathy, MLR values were higher in those diagnosed with lymphoma [10]. Additionally, high MLR values were found to be negative prognostic markers in ovarian cancer [13], and higher preoperative PLR values were observed in patients with early recurrences of pancreatic carcinoma [14].

In dogs, blood cell ratios were studied in various infectious and non-infectious inflammatory conditions, such as neoplasias, oral inflammatory and neoplastic pathologies, peritonitis, systemic inflammatory response and sepsis, inflammatory bowel disease, chronic enteropathy, inflammatory protein-losing enteropathy, acute pancreatitis, and pneumonia [15,16,17,18,19,20,21,22,23,24]. Among investigations of neoplasias, Rejec and colleagues (2017) [15] observed significantly higher NLR values in dogs with oropharyngeal tumors compared to those with periodontitis or healthy dogs. In dogs with mast cell tumors, higher NLR values were a negative prognostic factor for outcome [16]. Finally, increased values of NLR were significantly correlated with the risk of local recurrence in dogs with cutaneous perivascular wall tumors [22]. In studies about inflammatory conditions, the NLR values were higher in dogs affected by septic peritonitis and non-septic systemic inflammatory response syndrome (SIRS) compared to healthy dogs [17], but no difference was found between dogs with septic peritonitis and those with non-septic SIRS. Differently, lower NLR values were reported by Pierini and colleagues (2019, 2020) in dogs affected by sepsis compared to those with non-septic SIRS [18,20]. These authors studied various indexes and their prognostic value for risk of death in septic dogs, reporting persistently higher PLR and MLR values in non-survivors. Benvenuti and colleagues (2020) [19] evaluated the clinical significance of the NLR in dogs with inflammatory bowel disease, and they detected a positive correlation between the NLR and the canine chronic enteropathy clinical activity index (CCECAI). Additionally, they reported higher NLR values in dogs not responsive to immunomodulatory treatments [19]. In canine chronic enteropathy, higher NLR values were obtained in dogs with severe clinical disease than in dogs with mild forms [21]. Moreover, NLR was useful to distinguish between dogs with food-responsive enteropathy and dogs diagnosed with immunosuppressive-responsive or non-responsive enteropathies, with higher NLR values in the last two groups of dogs [21]. The NLR was also higher in dogs with inflammatory protein-losing enteropathy compared to healthy dogs [24]. In dogs with acute pancreatitis, NLR and PLR measures were significantly higher compared to healthy dogs; however, no relationship with disease severity was found [23]. In dogs with pneumonia, the NLR values did not differ between survivors and non-survivors [25].

Ratios between different circulating blood cells (NLR, MLR, and PLR) have been recently studied in cats with phlogistic [23,25,26] and neoplastic [27,28] diseases and proposed as diagnostic and prognostic markers for feline inflammatory and neoplastic conditions. In studies focusing on inflammatory conditions, NLR was higher in SIRS and septic cats compared to healthy cats, although it was not able to differentiate between SIRS and sepsis [25]. Interestingly, the NLR was significantly associated with mortality in both cats with SIRS and sepsis [25]. Similar to what is seen in dogs, significantly higher values of NLR and PLR were detected in cats with pancreatitis compared to healthy cats, and a significant increase of NLR and PLR was observed in cats with prolonged recovery [23]. Furthermore, cats with obstructive uropathy had significantly higher NLR and MLR than healthy cats, and PLR values differed according to the cause of the obstruction, being higher in cats with urethroliths than in those with idiopathic urethral obstruction or urethral plugs [26]. In investigations of cat neoplastic conditions, higher preoperative NLR values have been observed in cats with infiltrative forms of injection site sarcoma, fibrosarcomas, and local recurrence after surgical removal [27]. Higher NLR values were found to be a negative prognostic marker for disease-free survival, risk of recurrence, and tumor-related death in feline mammary carcinoma [28].

Although blood cell ratios have been evaluated in many feline pathologies, no information exists on the relationship between these ratios and markers of inflammation routinely measured in cats.

During inflammation, in response to the release of pro-inflammatory cytokines, the liver synthesizes the so-called acute phase proteins (APPs) that modulate the immune response by transporting molecules or by protecting tissues from excessive damage generated by inflammatory mediators [6]. The APPs used as markers of inflammation can be classified according to their increase (positive APPs, e.g., serum amyloid A) or decrease (negative APPs, e.g., albumin) during inflammation [6,29]. Moreover, APPs are classified according to their response to inflammation into “major”, “moderate”, and “minor”. Major APPs have a very low serum concentration that increases > 1000-fold after stimulation, with a peak observed at 24–48 h, and their concentrations decline rapidly during recovery. The increase of moderate APPs is 5- to 10-fold and peaks in 2–3 days, and the decrease is slower compared to major APPs. Minor APPs have a maximum two-fold increase from baseline values [29]. In cats, serum amyloid A (SAA) and α-1-acid glycoprotein (AGP) are major APPs, haptoglobin is a moderate APP, and C- reactive protein and ceruloplasmin are minor APPs [6]. Most APPs are globulins, and hyperglobulinemia also reflects the increased production of other proteins, particularly immunoglobulins, that can be observed in both inflammatory non-infectious [30,31] and infectious diseases [32,33,34,35,36,37,38,39]. Moreover, the albumin-to-globulin ratio (AGR), routinely reported in the biochemical profile, is considered a screening marker for dysproteinemia observed in feline inflammatory conditions [34,40,41]. Finally, as described above, neutrophil and lymphocyte abnormalities are seen in the circulating white blood cells of cats with inflammation. An increase in the number of band neutrophils and occasionally early precursors (left shift) may be observed, along with toxic changes (Döhle bodies, cytoplasmic basophilia, cytoplasmic vacuolation, and basophilic granulations) [4,6]. Moreover, the antigenic stimulation may induce increased size and cytoplasmic basophilia in lymphocytes (reactive lymphocytes) [4].

This retrospective preliminary study evaluated the relationships between some blood cell ratios (NLR, MLR, and PLR) and selected markers of inflammation (SAA, hypoalbuminemia, hyperglobulinemia, AGR, and the presence of leukocyte morphology alterations suggestive of inflammation) in cats, with the aim of verifying the hypothesis that blood cell ratio changes are indicative of phlogistic conditions.

## 2. Materials and Methods

### 2.1. Study’s Description and Characteristics of Cats

A retrospective review of the medical records of cats included in previous published studies was performed [36,42,43]. Considering the retrospective nature of the study, formal ethical approval was not necessary. Cats were sampled between 2012 and 2019 at four veterinary clinics located in Sicily (Ospedale Veterinario Universitario Didattico, Dipartimento di Scienze Veterinarie, Università di Messina, Messina; Ambulatorio Veterinario S. Lucia, Lipari, Messina) and Calabria (Clinica Veterinaria Camagna, Reggio Calabria; Ambulatorio Dr. Cardone, Gioia Tauro, Reggio Calabria) regions (Italy). Data about signalling and clinical history were recorded. From the database of these studied cats, some clinicopathological data were selected for the present study.

A total of 275 cats were enrolled in the study. Most cats came from Calabria (79.0%), with a higher percentage of female (64%) and outdoor (60.4%) cats evaluated. The median age was 24 months (min = 5 months, max = 228 months, 25^th^ percentile = 11 months, 75^th^ percentile = 60 months). Few pedigree (6.2%) (British Shorthair = 1, Chartreux = 3, Maine Coon = 8, Persian = 4, Ragdoll = 1) and domestic long-haired (6.5%) cats were included, while most cats were domestic short-haired (87.3%). The majority of enrolled cats were admitted for elective surgery (138), 65 for routine health checks, and 72 for various complaints. No information was available for seven cats. The diagnoses obtained with investigations performed in the 72 cats with complaints were: dermatitis (*n* = 10), trauma (*n* = 9), neoplasia (*n* = 8), acute gastroenteritis (*n* = 6), upper respiratory tract disease (*n* = 6), lower urinary tract disease (*n* = 5), chronic kidney disease (*n* = 3), stomatitis (*n* = 3), pyometra (*n* = 3), conjunctivitis (*n* = 2), unknown origin fever (*n* = 2), dystocia (*n* = 1), hepatic lipidosis (*n* = 1), and in 13 cats no diagnosis was made.

### 2.2. Clinicopathological Evaluation

The complete blood count (CBC) was performed using a laser hematology analyzer (IDEXX ProCyteDx^®^ Hematology Analyzer, IDEXX Laboratories, Westbrook, ME, USA) within two hours after collection, and reference intervals of the analyzer were used. Blood smears were prepared at the time of blood collection, stained with May-Grünwald-Giemsa stain (Merck KgaA, Darmstadt, Germany), and evaluated microscopically at oil immersion at ×1000 magnification [44] by a uniquely qualified operator (M.M.). Low platelet counts as well as any “smart flag” message reported by the analyzer about leukocyte or platelet counts (e.g., inability of the analyzer to make the count or inaccuracy of the analyzer count) were correspondingly confirmed or resolved after microscopic examination of blood smears. Blood smears were also examined for cell morphological abnormalities and to exclude thrombocytopenic samples from the statistical analysis when platelet clumps were observed. Leukocyte alterations suggestive of inflammation (LAI) were reported, and they included neutrophil left shift, cytoplasmic toxic changes in neutrophils, and reactive lymphocytes [4,45]. Left shift was reported when the number of band neutrophils was higher than 300/µL [46]. Cytoplasmic toxic changes in neutrophils included Döhle bodies (basophilic inclusions located in the peripheral cytoplasm), diffuse cytoplasmic basophilia, cytoplasmic vacuolation, and toxic granulations (magenta-staining granules) [45]. Reactive lymphocytes included large cells with broad, intensely basophilic cytoplasm [4,45]. Both neutrophil toxic changes and reactive lymphocytes were reported when they exceeded 5% of total neutrophils and lymphocytes, respectively [47]. The absolute counts of neutrophils (segmented and band neutrophils), monocytes, or platelets divided by the absolute count of lymphocytes were defined as NLR, MLR, and PLR, respectively. In serum samples, albumin, globulin, and their ratio (AGR) were measured by the Catalyst Dx ^®^ Chemistry Analyzer (IDEXX Laboratories, Westbrook, ME, USA) and SAA by a latex agglutination method (LZ-SAA, Eiken Chemical Co., Ltd., Tokyo, Japan) on an automated analyzer AU480 (Beckman Coulter, Brea, California, at the Department of Veterinary Medicine, Cambridge University, Cambridge, UK). Catalyst Dx^®^ Chemistry Analyzer reference intervals were used for albumin and globulins. Reference intervals for AGR and SAA were 0.45–1.19 [48] and <0.5 µg/mL (laboratory cut-off value), respectively.

### 2.3. Statistical Analysis

Statistical analysis was performed using GraphPad Prism version 7.0 for Windows (GraphPad Software, San Diego, CA, USA). The distribution of continuous variables was evaluated by the D’Agostino-Pearson omnibus normality test, and descriptive statistics were performed for all the investigated variables.

Mann-Whitney’s U-test was used to compare NLR, MLR, and PLR between cats with: (a) normal and increased SAA values; (b) normal and increased globulin values; (c) normal and decreased albumin values; (d) normal and decreased AGR values; (e) the presence and absence of LAI.

Spearman’s Rho test was used to measure the strength of the correlation between NLR, MLR, PLR, SAA, albumin, globulins, and AGR. Critical values of 0.118 (NLR and MLR) and 0.124 (PLR) for Spearman’s correlation coefficient were established, and the absolute magnitude of the observed *r_s_* was defined as follows: *r_s_* = 1: perfect correlation; 1 > *r_s_* ≥ 0.8: strong correlation; 0.8 > *r_s_* ≥ 0.4: moderate correlation; 0.4 > *r_s_* > 0.118: weak correlation; *r_s_* ≤ 0.118 (NLR and MLR) and ≤ 0.124 (PLR): no correlation [49].

Cats with normal values of SAA, globulins, albumin, AGR, and absence of LAI were selected for descriptive statistics of NLR, MLR, and PLR, and Mann-Whitney’s U-test was used to compare ratios of this group of cats to those with changes in single parameters (increases of SAA or globulins, decreases of albumin, and presence of LAI). No cat had a decrease in AGR as the only abnormality observed; therefore, this parameter was excluded from this statistical analysis.

Differences were considered significant if *p* values were <0.05.

## 3. Results

### Relationship between NLR, MLR, PLR, and Selected Markers of Inflammation

Total white blood cell counts, neutrophil, lymphocyte, monocyte, and platelet counts, selected inflammatory markers evaluated in the overall 275 cats, and the blood cell ratios calculated (NLR, MLR, and PLR) are reported in Table 1. In 26 cats, the PLR was not calculated due to the presence of platelet aggregates and the absence of the platelet count. In 17 cats, the evaluation of LAI was not made because of the poor quality of the blood smears.

A percentage of 22.5% (*n* = 58/258) of cats presented one or two signs of LAI [toxic neutrophils (*n* = 30); reactive lymphocytes and toxic neutrophils (*n* = 9); reactive lymphocytes (*n* = 9); left shift and toxic neutrophils (*n* = 5); left shift (*n* = 5)].

**Figure 1:**
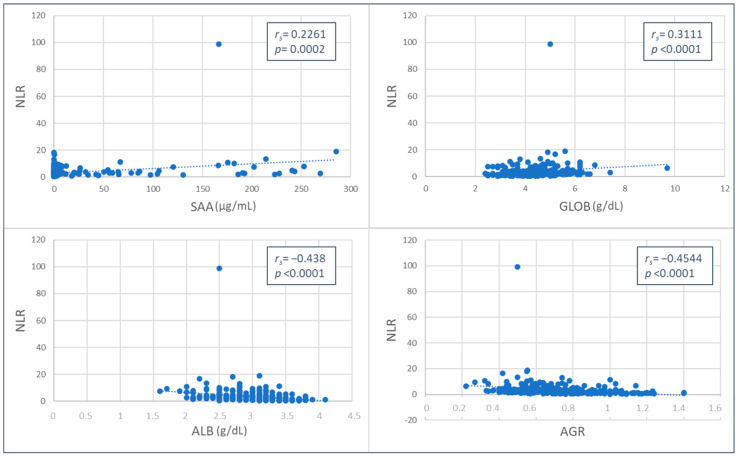
Spearman’s Rho correlation between neutrophil-to-lymphocyte ratio (NLR) and serum amyloid A (SAA), globulins (GLOB), albumin (ALB) values, and albumin-to-globulin ratio (AGR).

**Figure 2:**
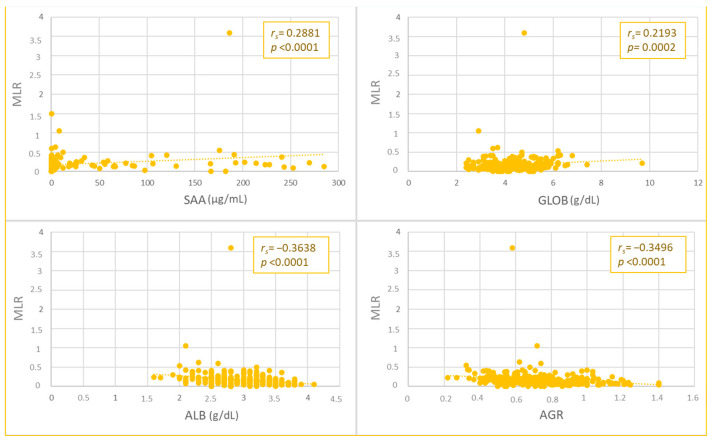
Spearman’s Rho correlation between monocyte-to-lymphocyte ratio (MLR) and serum amyloid A (SAA), globulins (GLOB), albumin (ALB) values, and albumin-to-globulin ratio (AGR).

The NLR and MLR values were weakly and positively correlated with SAA (NLR, *p* = 0.0002, *r_s_* = 0.2261; MLR, *p* < 0.0001, *r_s_* = 0.2881) and globulin concentrations (NLR, *p* < 0.0001, *r_s_* = 0.3111; MLR, *p* < 0.0002, *r_s_* = 0.2193), while a negative correlation was found with albumin (moderate for NLR, *p* < 0.0001, *r_s_* = −0.438; weak for MLR, *p* < 0.0001, *r_s_* = −0.3638) and AGR values (moderate for NLR, *p* < 0.0001, *r_s_* = −0.4544; weak for MLR, *p* < 0.0001, *r_s_* = −0.3496) (Figure 1 and Figure 2).

Moreover, higher values of NLR and MLR were found in cats with increased SAA and globulins and decreased albumin and AGR (Table 2). 

**Figure 3:**
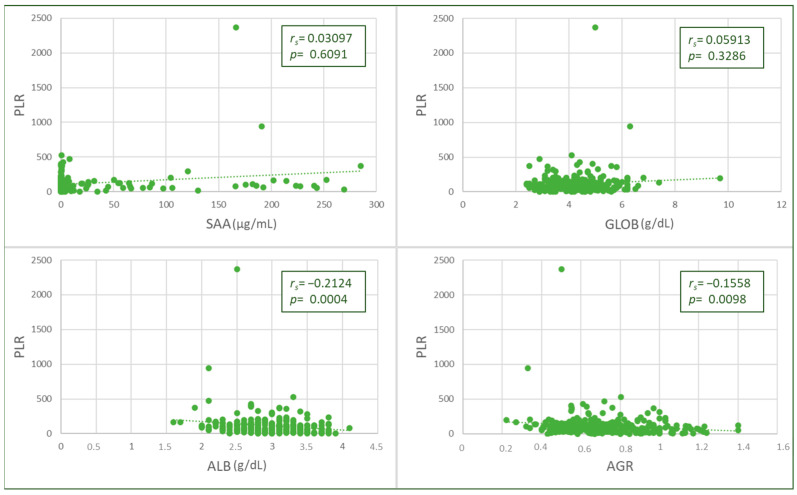
Spearman’s Rho correlation between platelet-to-lymphocyte ratio (PLR) and albumin (ALB) values and albumin-to-globulin ratio (AGR).

The PLR value was negatively and weakly correlated with albumin (*p* = 0.0006, *r_s_* = −0.2174) and AGR values (*p* = 0.0088, *r_s_* = −0.1658), and no significant correlations were found with SAA or globulins (Figure 3). Higher PLR values were found in cats with hypoalbuminemia (Table 2).

Higher NLR (*p* = 0.0001), MLR (*p* = 0.0236), and PLR (*p* = 0.0379) values were found in cats with LAI (Table 2). No significant differences in the PLR were found when cats with normal and increased SAA or globulin concentrations and normal and decreased AGR values were compared (Table 2).

Descriptive statistics of NLR (*n* = 93), MLR (*n* = 93), and PLR (*n* = 84) values in cats with normal SAA, globulin, albumin, AGR, and absence of LAI are reported in Table 3. Lower values of NLR and MLR were found in this group of cats compared to the 15 cats with the only increase in SAA (NLR *p* = 0.0008; MLR *p* = 0.0018) or the 30 cats with a decrease in albumin (NLR *p* < 0.0001; MLR *p* = 0.0231) concentrations. No significant differences were found when compared to the 14 cats with the only increase in globulins (NLR *p* = 0.2271; MLR *p* = 0.3243) or the 11 cats with the presence of LAI (NLR *p* = 0.4710; MLR *p* = 0.9418) (Table 3). The PLR was not different between cats with normal SAA, globulin, albumin, AGR values, and absence of LAI and cats with individually considered changes of these parameters [increase of SAA (*n* = 14; *p* = 0.2161) or globulins (*n* = 11; *p* = 0.5162), decreased albumin (*n* = 27; *p* = 0.0589), and presence of LAI (*n* = 11; *p* = 0.5619)] (Table 3).

## 4. Discussion

In this retrospective observational study, feline patients were studied with the purpose of exploring the relationship between blood cell ratios (NLR, MLR, and PLR) and selected markers of inflammation routinely evaluated in cats. Higher values of NLR and MLR were found both in cats with increased SAA and globulin concentrations and in those with decreased albumin and AGR values, while PLR calculations were higher in cats with hypoalbuminemia. In animals with LAI, all three ratios were higher. Additionally, NLR and MLR values correlated positively with the concentration of SAA and globulins. All the ratios studied (NLR, MLR, and PLR) showed a negative correlation with albumin concentration and AGR value. The strength of these correlations was weak to moderate, and this may depend on the different kinetics of parameters involved in the acute phase reaction and on the miscellaneous composition of the studied cats from a clinical point of view.

Leukocyte ratios were shown to be a better candidate as an additional marker of inflammation compared to the PLR. In fact, the cytokine-induced neuroendocrine activation associated with inflammation affects both leukocyte populations and APPs [6]. In particular, neutrophil demargination is the earliest mechanism responsible for the increase of mature circulating neutrophils, and it is much more important in cats as they have a higher proportion of neutrophils in the marginal pool compared to other species [4]. Reactive monocytosis occurs in cats under conditions where phagocytosis is required, such as infection and necrosis [4,5]. However, the increase in monocytes is generally modest, and monocytosis is observed less frequently than neutrophilia in infectious inflammation [5]. This can explain the lower strength of correlations observed for MLR compared to NLR. Inflammation is able to cause only a mild increase in the number of platelets in most cases, and this can justify the poorer performance of PLR in this study [50]. Interestingly, the best (negative) correlation was between NLR and the two negative acute-phase markers (albumin and AGR) studied, and these had a negative correlation with all three blood cell ratios. Therefore, in the population studied, the concomitant increase in the three blood cell ratios could be above all a marker of hypoalbuminemia. Albumin is a negative APP biomarker [51], but a decrease can be the consequence of multiple patho-mechanisms apart from decreased hepatic production [6]. It is not proven that hypoalbuminemia is caused by reduced liver synthesis in cats and that it is a real marker of inflammation [6], but decreased values are reported in a large number of feline inflammatory diseases [52]. The possibility of leakage of albumin from vessels to inflammation sites cannot be excluded in the studied cats and could have contributed to the present results. This latter patho-mechanism probably supported a negative correlation between NLR and hypoalbuminemia in dogs with inflammatory bowel disease [19], and higher NLR values were observed in dogs with chronic enteropathy [21]. However, the relationships of NLR, MLR, and partly PLR with the other markers of inflammation investigated support the utility of using these blood cell ratios in the diagnosis of inflammation in cats. In fact, SAA is a feline major positive APP [6,53], protecting tissues from excessive damage induced by inflammation and acting to regulate the inflammatory process and the immune response [54,55]. Hyperglobulinemia can be observed in both feline inflammatory [30,31] and infectious diseases [32,33,34,35,36,37,38,39]. Furthermore, in the serum protein electrophoretic evaluation, most of the positive APPs migrate as alpha- or beta-globulins, and immunoglobulins are beta- and gamma-globulins; thus, a globulin increase can give information about the presence of inflammatory and immune-mediated conditions [6]. The decrease in AGR is influenced by hypoalbuminemia and/or hyperglobulinemia and is considered a screening marker for feline diseases, with studies reporting its use in inflammatory [30,56], metabolic [57], and infectious diseases [6,34,58,59]. Left shift and toxic changes of neutrophils and reactive lymphocytes observed on blood smears are considered leukocyte abnormalities suggestive of inflammation, and we found that cats presenting any of these changes had higher values of NLR, MLR, and PLR. We included the analysis of these morphological changes in this study because they are linked to mechanisms involved in inflammation. In fact, the presence of immature neutrophils (left shift) indicates that the stimulus for the release of neutrophils from bone marrow is greater than the ability to release mature neutrophils from bone marrow stores [4,45]. Moreover, toxic changes in neutrophils are caused by cytokines produced in strong inflammatory conditions [4,6,45]. Toxic neutrophils can be observed before quantitative changes in the leukogram and may be the only hematological alteration indicative of inflammation. Finally, reactive lymphocytes arise from a response of these cells to antigenic stimulation [4,45,60].

In the group of cats with no changes in parameters indicative of inflammation, the maximum values of NLR, MLR, and PLR were 11.25, 0.42, and 528.3, respectively (Table 3). As the values of NLR and MLR of these cats were significantly lower when compared with those of cats with increased SAA or hypoalbuminemia, we consider these as possible cut-off values for cats with no inflammation. There are no studies investigating cut-off values or reference ranges of blood cell ratios in cats, while many studies were recently performed in humans and very few in dogs. In dogs, two studies proposed reference intervals for healthy control animals, but they were obtained with different statistical methodologies, and their upper limits largely differed from each other: 10.91 [17] and 4.1 [21]. Mutz et al. (2015) set a dog NLR cut-off value by simply dividing the lower end of the normal reference range for absolute neutrophil count by the lower end of the normal reference range for absolute lymphocyte count [61]. However, cut-off values for cats have to be better established by appropriate analysis of larger numbers of data from cats of different ages, breeds, and sexes and by considering healthy animals and cats with both inflammatory and non-inflammatory conditions.

We considered these data preliminary because they were obtained in a retrospective study, and some limitations are due to this type of study. In fact, only cross-sectional data from cats admitted to veterinary clinics for various reasons was available. This means that in the cats with various complaints, the time of blood sampling may have occurred at varied time distances with respect to the triggering event. Moreover, the data available were mostly from apparently healthy cats (admitted for elective surgery and routine health checks), and the clinicopathological investigation in those admitted for complaints (26.8%) was not homogenous. In particular, additional causes of hypoalbuminemia apart from inflammation, such as malnutrition, liver failure, protein-losing nephropathies, and enteropathies, could not be excluded in all affected cats [52]. Due to these sample biases, we could not compare the values of the studied ratios between healthy cats and cats with non-inflammatory and inflammatory conditions. However, we compared the blood cell ratio values of cats with all the studied acute-phase parameters within the reference range and with no signs of LAI with those of individuals with single abnormalities suggestive of inflammation. Finally, we considered in this study the unique feline major APP (SAA); however, other APPs have different biological functions and kinetics and are currently available from commercial laboratories. This is the case with haptoglobin and AGP, and their relationship with blood cell ratios could be different.

It should also be mentioned that blood cell ratios themselves have limits. For instance, in cases of severe inflammation, neutropenia [6] may occur, but an NLR increase would not occur. This means that blood cell ratio values should always be considered as part of the overall CBC report, and clinicians should take into consideration the comments on the blood smears, cell morphology, and cell counts.

We were driven to explore in cats the relationships of some acute-phase markers with blood cell ratios easily obtained in the CBC report because this is a baseline hematological test always included in the minimal clinicopathological evaluation database of patients. Any additional clinical information obtained from measured parameters of the CBC, such as blood cell ratios, can provide useful information without requiring additional costs for owners and possibly reducing the volume of blood required for other tests, which is a big plus in cats. Moreover, veterinary clinics are commonly equipped with in-house CBC analyzers, and blood cell ratios can be obtained within minutes of collection in emergencies.

## 5. Conclusions

The relationships found among some AP markers and NLR, MLR, and PLR values show that these ratios are influenced by inflammatory patho-mechanisms in cats. Furthermore, they provide the preliminary rationale for undertaking prospective and longitudinal studies for the validation of these blood cell ratios as additional cost-effective diagnostic and prognostic markers of inflammation in feline patients.

## Figures and Tables

**Table 1:** Reference intervals (RI) and descriptive statistics of parameter values studied, number (*n*) and percentage (%) of cats with abnormalities in these parameters.

Parameter [Rl]	Median (Min–Max) [25^th^ Percentile–75^th^ Percentile]	Abnormality [*n* (%)]
**CBC**		
White blood cells [2.87–17.02 K/µL]	11.82 (0.38–42.99)	Leukocytosis [65 (23.6)]
	[25^th^: 8.1; 75^th^: 16.8]	Leukopenia [2 (0.7)]
Neutrophils [2.30–10.29 K/µL]	6.9 (0.2–40.77)	Neutrophilia [78 (28.4)]
	[25^th^: 4.4; 75^th^: 11.0]	Neutropenia [11 (4)]
Lymphocytes [0.92–6.88 K/µL]	3.1 (0.07–13.14)	Lymphocytosis [16 (5.8)]
	[25^th^: 2.1; 75^th^: 4.2]	Lymphopenia [12 (4.4)]
Monocytes [0.05–0.67 K/µL]	0.39 (0–5.14)	Monocytosis [48 (17.5)]
	[25^th^: 0.3; 75^th^: 0.6]	
Platelets [151–600 K/µL]	261 (0–1051)	Thrombocytosis [6 (2.2)]
	[25^th^: 151; 75^th^: 356]	Thrombocytopenia [57 (20.7)]
**Serological inflammatory markers**		
Serum amyloid A [<0.5 µg/mL]	0.4 (0.01–285.1)	Increased value [80 (29.1)]
	[25^th^: 0.1; 75^th^: 1]	
Albumin [2.3–3.9 g/dL]	3 (1.6–4.1) [25^th^: 2.7; 75^th^: 3.3]	Hypoalbuminemia [107 (38.9)]
Globulins [2.8–5.1 g/dL]	4.2 (2.4–9.7)	Hyperglobulinemia [81 (29.5)]
	[25^th^: 3.6; 75^th^: 4.8]	
AGR [0.45- 1.19]	0.7 (0.2–1.4)	Decreased value [20 (7.3)]
	[25^th^: 0.6; 75^th^: 0.9]	
**Blood cells ratios**		
NLR	2.4 (0.3–99.0)	ne
	[25^th^: 1.5; 75^th^: 4.0]	
MLR	0.1 (0–3.6)	
	[25^th^: 0.1; 75^th^: 0.2]	
PLR	92.6 (0–2368)	ne
	[25^th^: 51.5; 75^th^: 143.4]	

CBC = complete blood count; AGR = albumin-to-globulin ratio; ne = not evaluable; NLR = neutrophil-to-lymphocyte ratio; MLR = monocyte-to-lymphocyte ratio; PLR = platelet-to-lymphocyte ratio.

**Table 2:** Comparisons of median values of neutrophil-to-lymphocyte (NLR), monocyte-to-lymphocyte (MLR), and platelet-to-lymphocyte (PLR) ratios among cats with normal and decreased (albumin, AGR) or increased (SAA, globulin) values, and between cats with LAI and those with no LAI. For each parameter, the number (*n*) of cats with normal, decreased (albumin, AGR), or increased (SAA, globulin) values and the presence of LAI was indicated.

	SAA		ALB		GLOB		AGR		LAI	
**NLR**	*p* < 0.0001		*p* < 0.0001		*p* < 0.0001		*p* = 0.0006		*p* = 0.0001	
	**Normal**	**Increased**	**Normal**	**Decreased**	**Normal**	**Increased**	**Normal**	**Decreased**	**Absence**	**Presence**
	(*n* = 195)	(*n* = 80)	(*n* = 168)	(*n* = 107)	(*n* = 194)	(*n* = 81)	(*n* = 255)	(*n* = 20)	(*n* = 200)	(*n* = 58)
	Median: 2.00	Median: 3.42	Median: 1.78	Median: 3.19	Median: 2.02	Median: 3.015	Median: 2.29	Median: 3.96	Median: 2.14	Median: 3.72
	Min: 0.35	Min: 0.43	Min: 0.35	Min: 0.43	Min: 0.35	Min: 0.51	Min: 0.35	Min: 1.92	Min: 0.41	Min: 0.46
	Max: 18.18	Max: 98.97	Max: 18.83	Max: 98.97	Max: 13.14	Max: 98.97	Max: 98.97	Max: 16.62	Max: 18.83	Max: 98.97
	25^th^: 1.19	25^th^: 2.05	25^th^: 1.09	25^th^: 2.16	25^th^: 1.23	25^th^: 1.98	25^th^: 1.38	25^th^: 2.35	25^th^: 1.38	25^th^: 2.10
	75^th^: 3.68	75^th^: 6.59	75^th^: 3.20	75^th^: 4.55	75^th^: 3.65	75^th^: 5.13	75^th^: 3.84	75^th^: 8.07	75^th^: 3.68	75^th^: 7.47
**LR**	*p* < 0.0001		*p* = 0.0008		*p* = 0.0003		*p* < 0.0001		*p* = 0.0236	
	**Normal**	**Increased**	**Normal**	**Decreased**	**Normal**	**Increased**	**Normal**	**Decreased**	**Absence**	**Presence**
	(*n* = 195)	(*n* = 80)	(*n* = 168)	(*n* = 107)	(*n* = 194)	(*n* = 81)	(*n* = 255)	(*n* = 20)	(*n* = 200)	(*n* = 58)
	Median: 0.12	Median: 0.19	Median: 0.13	Median: 0.17	Median: 0.13	Median: 0.18	Median: 0.14	Median: 0.22	Median: 0.14	Median: 0.18
	Min: 0	Min: 0	Min: 0	Min: 0	Min: 0	Min: 0	Min: 0	Min: 0.08	Min: 0	Min: 0
	Max: 0.59	Max: 3.59	Max: 0.5	Max: 3.59	Max: 1.06	Max: 3.59	Max: 3.59	Max: 0.54	Max: 0.59	Max: 3.59
	25^th^: 0.08	25^th^: 0.14	25^th^: 0.07	25^th^: 0.10	25^th^: 0.08	25^th^: 0.12	25^th^: 0.08	25^th^: 0.18	25^th^: 0.09	25^th^: 0.09
	75^th^: 0.19	75^th^: 0.25	75^th^: 0.19	75^th^: 0.24	75^th^: 0.19	75^th^: 0.25	75^th^: 0.19	75^th^: 0.38	75^th^: 0.19	75^th^: 0.27
**PLR** ^	*p* = 0.7843		*p* = 0.0168		*p* = 0.0539		*p* = 0.1903		*p* = 0.0439	
	**Normal**	**Increased**	**Normal**	**Decreased**	**Normal**	**Increased**	**Normal**	**Decreased**	**Absence**	**Presence**
	(*n* = 175)	(*n* = 74)	(*n* = 153)	(*n* = 96)	(*n* = 179)	(*n* = 70)	(*n* = 230)	(*n* = 19)	(*n* = 179)	(*n* = 53)
	Median: 97.15	Median: 90.13	Median: 83.88	Median: 109.3	Median: 87.16	Median: 109.8	Median: 89.96	Median: 115.6	Median: 84.83	Median: 116.5
	Min: 0	Min: 0	Min:0	Min: 0	Min: 0	Min: 0	Min:0	Min: 0	Min:0	Min:0
	Max: 528.3	Max: 2368	Max: 528.3	Max: 2368	Max: 528.3	Max: 2368	Max: 2368	Max: 942.9	Max: 942.9	Max: 2368
	25^th^: 47.95	25^th^: 55.32	25^th^: 40.79	25^th^: 62.25	25^th^: 47.95	25^th^: 60.44	25^th^: 49.61	25^th^: 63.41	25^th^: 47.54	25^th^: 75.97
	75^th^: 143.0	75^th^: 150.0	75^th^: 133.5	75^th^: 154.9	75^th^: 134.0	75^th^: 162.8	75^th^: 142.7	75^th^: 169.7	75^th^: 137.5	75^th^: 166.8

AGR = albumin-to-globulin ratio; ALB = albumin; GLOB = globulins; LAI = leukocyte abnormalities suggestive of inflammation; SAA = serum amyloid A. Max = maximum; Min = minimum; 25^th^ = 25^th^ percentile; 75^th^ = 75^th^ percentile. PLR ^ = in 26 cats, the PLR was not calculated due to the presence of platelet aggregates and the absence of the platelet count

**Table 3:** Descriptive statistics of values of neutrophil-to-lymphocyte (NLR), monocyte-to-lymphocyte (MLR) and platelet-to-lymphocyte (PLR) ratios from 93 (NLR and MLR) and 84 (PLR) cats considered with no inflammation (No-I) because of normal values of serum amyloid A (SAA), globulins (GLOB), albumin (ALB), albumin-to-globulin ratio (AGR), and absence of leukocyte abnormalities suggestive of inflammation (LAI) and of cats with signs of inflammation consisting in increased SAA (∧SAA; number of cats: NLR and MLR = 15; PLR = 14) or GLOB (∧GLOB; number of cats: NLR and MLR = 14; PLR = 11) concentrations, decreased ALB (∨ALB; number of cats: NLR and MLR = 30; PLR = 27) values, presence of LAI (+LAI; number of cats: NLR, MLR and PLR = 11). No cats presented reduced AGR as a single abnormality.

	NLR					MLR					PLR				
	No-I	∧SAA	∨ALB	∧GLOB	+LAI	No-I	∧SAA	∨ALB	∧GLOB	+LAI	No-I	∧SAA	∨ALB	∧GLOB	+LAI
Min.	0.41	1.29	0.78	0.51	0.46	0	0.09	0	0.04	0.04	0.16	2.67	30.77	37.98	0
25^th^	1.02	1.65	1.93	1.07	0.69	0.07	0.14	0.09	0.07	0.07	42.36	17.11	61.99	40.19	24.42
Median	1.52	2.68	3.19	1.67	1.08	0.11	0.17	0.14	0.15	0.09	81.76	66.97	109.3	87.75	71.39
75^th^	2.57	6.77	3.87	4.97	5.15	0.16	0.24	0.23	0.21	0.18	129	110.3	160.9	227.5	119.1
Max.	11.25	11.03	8.91	9.24	10.78	0.42	0.5	0.59	0.35	0.3	528.3	301.9	390.1	355.8	231.9

Max. = maximum; Min. = minimum; 25^th^ = 25^th^ percentile; 75^th^ = 75^th^ percentile.

## Data Availability

The data set analyzed for the current study is available from the corresponding author upon reasonable request.
